# Piecewise Structural Equation Model (SEM) Disentangles the Environmental Conditions Favoring Diatom Diazotroph Associations (DDAs) in the Western Tropical North Atlantic (WTNA)

**DOI:** 10.3389/fmicb.2017.00810

**Published:** 2017-05-09

**Authors:** Marcus Stenegren, Carlo Berg, Cory C. Padilla, Stefan-Sebastian David, Joseph P. Montoya, Patricia L. Yager, Rachel A. Foster

**Affiliations:** ^1^Department of Ecology, Environment and Plant Sciences, Stockholm UniversityStockholm, Sweden; ^2^Science for Life Laboratory, Department of Biology and Environmental Science, Linnaeus UniversityKalmar, Sweden; ^3^School of Biology, Georgia Institute of Technology, AtlantaGA, USA; ^4^Max Planck Institute for Biophysical ChemistryGöttingen, Germany; ^5^Max Planck Institute for Marine MicrobiologyBremen, Germany; ^6^Department of Marine Sciences, University of Georgia, AthensGA, USA; ^7^Ocean Sciences, University of California, Santa CruzSanta Cruz, CA, USA

**Keywords:** symbioses, cyanobiont, diatoms, nifH, Amazon, DDAs, piecewise SEM

## Abstract

Diatom diazotroph associations (DDAs) are important components in the world’s oceans, especially in the western tropical north Atlantic (WTNA), where blooms have a significant impact on carbon and nitrogen cycling. However, drivers of their abundances and distribution patterns remain unknown. Here, we examined abundance and distribution patterns for two DDA populations in relation to the Amazon River (AR) plume in the WTNA. Quantitative PCR assays, targeting two DDAs (het-1 and het-2) by their symbiont’s *nifH* gene, served as input in a piecewise structural equation model (SEM). Collections were made during high (spring 2010) and low (fall 2011) flow discharges of the AR. The distributions of dissolved nutrients, chlorophyll-*a*, and DDAs showed coherent patterns indicative of areas influenced by the AR. A symbiotic *Hemiaulus hauckii-Richelia* (het-2) bloom (>10^6^ cells L^-1^) occurred during higher discharge of the AR and was coincident with mesohaline to oceanic (30–35) sea surface salinities (SSS), and regions devoid of dissolved inorganic nitrogen (DIN), low concentrations of both DIP (>0.1 μmol L^-1^) and Si (>1.0 μmol L^-1^). The *Richelia* (het-1) associated with *Rhizosolenia* was only present in 2010 and at lower densities (10-1.76 × 10^5^ *nifH* copies L^-1^) than het-2 and limited to regions of oceanic SSS (>36). The het-2 symbiont detected in 2011 was associated with *H. membranaceus* (>10^3^ *nifH* copies L^-1^) and were restricted to regions with mesohaline SSS (31.8–34.3), immeasurable DIN, moderate DIP (0.1–0.60 μmol L^-1^) and higher Si (4.19–22.1 μmol L^-1^). The piecewise SEM identified a profound direct negative effect of turbidity on the het-2 abundance in spring 2010, while DIP and water turbidity had a more positive influence in fall 2011, corroborating our observations of DDAs at subsurface maximas. We also found a striking difference in the influence of salinity on DDA symbionts suggesting a niche differentiation and preferences in oceanic and mesohaline salinities by het-1 and het-2, respectively. The use of the piecewise SEM to disentangle the complex and concomitant hydrography of the WTNA acting on two biogeochemically relevant populations was novel and underscores its use to predict conditions favoring abundance and distributions of microbial populations.

## Introduction

*Richelia intracellularis* is one of the few heterocystous cyanobacteria commonly found in the oligotrophic oceans and are considered an important source of new nitrogen (N) and primary production (Mague et al., 1974; Venrick, 1974). *Richelia* forms intimate, inconspicuous, and highly specific relationships with several diatom genera, including *Rhizosolenia* and *Hemiaulus* (Janson et al., 1999; Foster and Zehr, 2006). The diatom symbioses are widespread in distribution, fragile, hard to collect and recognize, and therefore difficult to study. The diatoms and their respective diazotrophic partners are often referred to as Diatom Diazotroph Associations, or DDAs.

The western tropical North Atlantic (WTNA) near the Amazon and Orinoco river plumes is an ideal location to study the presence, distribution, and activity of symbiotic diatoms, since large and expansive blooms of the DDA, *Hemiaulus-Richelia*, are consistently observed (Villareal, 1994; Carpenter et al., 1999; Foster et al., 2007; Subramaniam et al., 2008; Goes et al., 2014). The area is largely influenced by both fluvial and atmospheric deposition of dissolved nutrients (e.g., nitrate, phosphate, silicate) and trace metals (e.g., iron) (DeMaster et al., 1983; Tovar-Sanchez and Sañudo-Wilhelmy, 2011). In addition, the surface euphotic zone of the WTNA is also affected by eddy upwelling and horizontal advection (Muller-Karger et al., 1988, 1995; Longhurst, 1993), and is therefore also a dynamic region to study the influence of various environmental parameters on a planktonic community. The influence of the rivers is far-reaching and seasonal, where within the freshwater lenses, distinct phytoplankton populations and enhanced nutrient concentrations can be observed at distances greater than 1,600 km away from the river mouths (Borstad, 1982a,b; Muller-Karger et al., 1988, 1995).

Earlier studies in the WTNA have also reported it as an area of both intense diazotrophy and carbon sequestration. For example, a widespread bloom of *Hemiaulus-Richelia* symbioses combined with low densities of the colonial filamentous *Trichodesmium* spp. were estimated to provide nearly 0.5 Tg N to the surface ocean within a 10 day period, an estimate of new N that far exceeds the calculated flux of nitrate from below the euphotic (Carpenter et al., 1999). Later, Subramaniam et al. (2008) estimated that *Hemialus-Richelia* blooms sequester nearly 20 Tg carbon (C) annually to the deep ocean. Similarly, in the Subtropical North Pacific gyre, elevated and annually recurrent summer time export is largely mediated by DDAs (Karl et al., 2012). High abundances of DDAs have also been reported from other river plumes, e.g., Congo and Niger as well as the South China Sea (Foster et al., 2009; Bombar et al., 2011), and most recently the *H. hauckii* with associated *R. intracellularis* was reported 84 out of the 145 oceanic stations sampled during the global Malaspina-2010 survey (December 2010–July 2011) with a bloom recorded off the Brazilian coast (Estrada et al., 2015). Thus the abundance and overall importance of DDA blooms to C and N cycling is well known, however, the driver of DDA abundances is little known.

In the last decade abundances of various diazotrophs are often estimated by their nitrogenase (*nifH*) gene and quantitative polymerase chain reaction (qPCR) assays. For the DDAs, abundances are reported as het-1 and het-2 *nifH* genes, which refers to the *R. intracellularis* symbiont of *Rhizosolenia* spp. and *Hemiaulus* spp., respectively (Foster and Zehr, 2006). The het-2 assay does not distinguish between the *R. intracellularis* associated with *H. hauckii* and *H. membranaceus*. Moreover some qPCR datasets have been used as input for model-based approaches to estimate N_2_ fixation, growth rates, distribution, and seasonality of diazotrophs (e.g., Goebel et al., 2007, 2008, 2010; Luo et al., 2012). However, seldom are the DDAs featured. A rather new approach to predicting distribution and constraints on abundances of organisms, including microorganisms, is the use of structural equation modeling (SEM) (Duffy et al., 2015; Jing et al., 2015; Lefcheck and Duffy, 2015). Multivariate SEM (Shipley, 2000, 2009) is a type of path analysis, which differs from traditional models as it incorporates and tests hypothesized causality. Additionally any parameter, e.g., temperature, can act as both a predictor and a response variable in the model pathways, thus, allowing for the identification and quantification of indirect and cascading effects in a system on a particular target. Here, we used SEM to understand better the influence of the Amazon River (AR) Plume on the DDAs.

Given the biogeochemical significance of symbiotic diatoms, the primary objective of our investigation was to determine the abundance and distribution patterns for DDAs in the WTNA. Our work used the contrasting hydrographic conditions derived from seasonal (summer 2010 and fall 2011) differences in the AR discharge to determine conditions that influence the abundance, distribution and activity of DDAs. Subsequently the abundance of the DDAs were used at input in a piecewise SEM (Lefcheck, 2016) to identify the direct and indirect effects of environmental variable(s) influencing the two observed DDA populations. The results provide new information on drivers of species distribution in an area of the world’s ocean known for high carbon sequestration (Subramaniam et al., 2008).

## Materials and Methods

### Sample Collection

**FIGURE 1:**
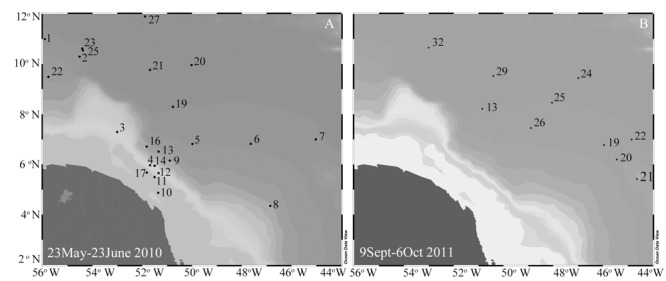
**Map of the WTNA with station locations for field expeditions in 2010 (A)** and 2011 **(B)**.

Expeditions to the WTNA were conducted May 23–June 23, 2010 and September 4–October 6, 2011 on board the R/V Knorr and R/V Melville, respectively. **Figure 1** shows the cruise tracks and station locations for sample collections during the field expeditions. Sampling for nucleic acids were limited to the second leg (September 9–October 6, 2011) of 2011. Seawater was collected from discrete depths using Niskin bottles (12 or 24 L) arranged on a Conductivity Temperature Depth (CTD) rosette. The suite of parameters recorded at the time of nucleic acid collection and used in the statistical analyses (see below) is summarized in Supplementary Table S1.

#### Nutrient Analyses

Samples for measurement of the concentrations of dissolved inorganic nitrogen (DIN: nitrate + nitrite), dissolved inorganic phosphate (DIP), and silicate (Si) were collected directly from the ctd-rosette and were measured at sea using standard colorimetric methods (Grasshoff et al., 1983) on a Lachat QuikChem 8000 FIA system (Knap et al., 1994). Samples were generally analyzed within two to 3 h of collection, and aliquots were frozen for reanalysis ashore if needed. Reanalyses were performed within 6–8 months of collection. The detection limits for DIN, DIP, and Si were 0.1, 0.05, and 0.05 μmole L^-1^, respectively.

### Microscopy Observations

Cells for microscopy observations were collected directly from the CTD at the same or hydrographically similar depths as water collected for DNA samples, although at different times. The microscopy samples were always collected at local noon, while the CTD casts for the nucleic acid samples varied from early morning until late afternoon. The entire contents of the Niskin were gravity filtered onto a 47 mm diameter Poretics (Millipore) membrane filter with a pore size of 10 μm. Gravity-filtration time varied from 30 min to 2 h. If the filter clogged by 2 h, the remaining volume in the Niskin was measured using a graduated cylinder, and the volume filtered was noted. The filter was mounted onto an oversize microscope slide (75 mm × 50 mm × 1 mm) and examined at 400X under a Zeiss Axioskop Epifluorescence microscope (Zeiss, Berlin, Germany). Phycoerythrin and chlorophyll *a* (Chl *a*) was used to identify the symbiotic *Richelia* and the phytoplankton community by epifluorescence microscopy using green (510–560 nm) and blue (450–490) nm excitation wavelengths, respectively. The hosts of the DDAs were identified based on cell morphology. Qualitative observations of cell integrity for the DDAs, and general composition of the phytoplankton community were recorded.

#### DNA Collection

Seawater was collected directly into bleach-rinsed 2.5 L polycarbonate bottles and immediately filtered using a peristaltic pump (Cole-Parmer, Vernon Hills, IL, USA) through a 0.2 μm pore size Supor filter (Pall Corporation) held within a 25 mm diameter Swinnex filter holder (Millipore, Billerica, MA, USA). The volume of seawater filtered was 1–2.5 L. After filtration, the filters were removed and placed in sterilized 2 mL bead beater tubes (Biospec) containing 0.1mm and 0.5 mm glass bead mixture (Biospec Products), then frozen in liquid N_2_ and stored at -80°C until further processed.

### DNA Extraction and Quantitative PCR

Nucleic acids were extracted using the modified DNAeasy plant kit (Qiagen) method described in Moisander et al. (2008) and the final elution volume was 70 μL. The *Richelia* specific TaqMan (Applied Biosystems) PCR primers and probes described in Church et al. (2005) and Foster et al. (2007) were used to quantify the abundance of het-1 and het-2. The TaqMan PCRs were conducted in a GeneAmp 9700 (Applied Biosystems) or MiniOpticon (Biorad) sequence detection systems with assays and conditions as previously described (Foster et al., 2007). Briefly, the following parameters apply: 50°C for 2 min, 95°C for 10 min, and 45 cycles of 95°C for 15 s followed by 60°C for 1 min. Reactions were performed in quadruplicate with the fourth replicate used to estimate the reaction efficiency (see below). Two microliter of 5 kDa filtered nuclease free water was used for the no template controls (NTCs). No *nifH* copies for either het group were detected in the NTCs. The detection limit of the assay is between 1 and 10 *nifH* copies.

Gene copy abundances were calculated from the mean *C*_t_ value of the three replicates and the standard curve for the appropriate primer and probe set (see below). Samples where one or two of the three replicates produced an amplification signal, were noted as detectable, but not quantifiable (dnq). The *nifH* gene copy abundances have been normalized to copy numbers per cell in the *Richelia* trichome by dividing by 5 and 4 for het-1 and het-2, respectively, as these were the number of cells observed by microscopy in the respective symbiont’s trichome.

### Standard Curves and PCR Efficiency

For each primer and probe set, duplicate standard curves were made from 10-fold dilution series ranging from 1 to 10^8^ gene copies per reaction. The standard curves were made from linearized plasmids containing the *nifH* gene. Regression analyses of the number of cycles were analyzed in Excel. The PCR efficiency for each sample was determined as previously described by Short et al. (2004).

### Statistical Analysis

Multivariate analysis in the form of piecewise SEM was conducted in the software R^1^ using the packages ‘NLME,’ ‘lavaan,’ and ‘piecewiseSEM’ (Lefcheck, 2016). An initial spearman’s rank correlation on the qPCR data was performed using IBM SPSS (ver. 23), which covered correlations of all parameters measured in the system and investigated in the piecewise SEM model.

The results of the spearman rank correlation combined with previous knowledge on the investigated system were used to formulate hypotheses on pathways of interaction between parameters in the model, where all parameters could act as both predictor and response variables. The hypotheses acted as a framework when designing and optimizing the piecewise SEM to fit the generated data. All measured parameters were included in the framework, but not all of them, due to statistical non-significance (*p* > 0.05), were still part of the model after optimization. Additionally, station and depth were considered random factor variables in the model.

Built into the ‘piecewise SEM’ package is a ‘missing pathways’ command, which provides information, supported by statistical significance to improve the SEM model. Thus, the piecewise SEM model is stepwise and modified to account for missing or incomplete pathways between response and predictor variables for each general linear mixed effects model that makes up the piecewise SEM model. The missing pathways were then tested in parallel with evaluation of the Akaike Information Criterion (AIC), which estimates the robustness of the current model compared to other models of the same dataset, but generated with different pathways (Shipley, 2013). If the tested pathways were statistically significant (*p* < 0.05) and generated a lower AIC score, they were included and the model was further optimized to account for more variation. Subsequently, non-significant parameters were excluded from the model. It is important to note that AIC in itself does not explain variation but is rather a means to compare models derived from the same dataset.

When the final optimized model was procured (lowest AIC score with the most variables included) chi-square statistics were run to evaluate the model goodness-of-fit (Shipley, 2009; Lefcheck, 2016). If the chi-square was statistically non-significant (*p* > 0.05) the model was a good fit to the data. In addition to the goodness-of-fit for the model as a whole, an *R*^2^ value was calculated for each general linear mixed effects model. The *R*^2^ value is a measure of the variation in the data explained by a general linear mixed effects model for a particular pathway.

Finally, in addition to the direction of the effects found between variables in the piecewise SEM model, a pathway table, with estimate values for all direct effects was reported. The effect estimates were used to calculate and compare the strengths of direct and indirect effects between variables in the system. Indirect effects were described as a predictor variable (*Y*) having an effect on a response variable (*X*) through a simultaneous response and predictor variable (*Z*) and is represented by the following:

(1) $$\begin{array}{c} Y\;\Rightarrow \;Z\;\Rightarrow \;X \end{array}$$

For example:

(2) $$\begin{array}{c} \mathrm{Depth}\;\Rightarrow \;\mathrm{DIP}\;\Rightarrow \;het-1 \end{array}$$

(3) $$\begin{array}{c} 0.43\;\times \;0.28\;=\;0.12 \end{array}$$

The arrows (1–2) represent the effect estimate values that are multiplied (3) to procure the strength and direction (positive or negative) of the indirect effect of predictor variable *Y* on response variable *X*. In equation 3, the numbers are derived from SEM of 2010 dataset.

## Results

### Hydrographic Conditions and Nutrient Concentrations

**FIGURE 2:**
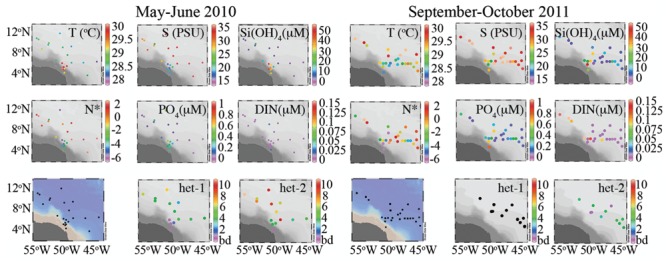
**Summary of surface hydrographic conditions (0–5 m) and depth integrated *nifH* gene copy abundance for the diatom symbioses in 2010 and 2011 expeditions.** In 2011, the het-1 (*Richelia* associated with *Rhizosolenia*) was below detection.

Following an earlier classification scheme based on sea surface salinities (SSS), three salinity categories were defined: ‘low’ SSS containing stations with SSS < 30, ‘mesohaline’ stations have SSS between 30 and 35, and stations with SSS > 35 were classified as ‘oceanic’ (Foster et al., 2007). A summary of the stations and nutrient concentrations for the three categories is provided in **Table 1**, and the corresponding station locations and surface nutrient concentrations are shown in **Figures 1, 2**, respectively.

**Table 1:** Summary of nutrient concentration ranges as a function of sea surface (<4 m) salinity (SSS) categories (low, meso, high) for expeditions to the Western Tropical North Atlantic (WTNA) in 2010 and 2011.

SSS	Stations	DIN (μmol L^-1^)	DIP (μmol L^-1^)	Silicate (mmols L^-1^)
Low (16.4–29.4)	**2010**: 9^∗†^, 11–13, 14^∗†^, 16, 17, 23, 4, 9, 10, 14	bd 0.02–1.46	0.21–0.59	21.5–56.6
(28.5–29.7)	**2011**: 6, 11–13^∗^, 17, 5, 6^†^, 7	bd 0.09–0.43	0.09–0.41	6.29–40.3
			0.24–1.02	19.3–42.1
			0.18–0.75	20.4–36.9
Meso (30.2–34.9)	**2010**: 2–3, 9^∗^, 19, 22, 25, 1, 21	bd 0.02–0.03	bd–0.19	2.19–17.0
(31.8–34.3)	**2011**: 8, 13^∗†^, 16, 19^†^, 20–25, 26^∗^, 29, 1–3, 9^∗^, 13^∗†^, 19^†^, 21^†^, 32	bd 0.06–0.93	0.06–0.11	5.61–10.7
			0.10–0.60	4.19–22.1
			0.07–0.59	4.35–17.68
High (35.2–36.0)	**2010**: 5–8, 20, 6^†^, 7^†^, 27	bd 0.02–0.11	bd–0.70	0.79–2.76
(35.2–36.1)	**2011**: 10^†^, 13^∗^–15, 26^∗†^, 9^∗^, 10^†^, 26^∗†^	bd 0.11	0.03–0.11	0.78–0.80
			0.05–0.23	0.88–5.99
			0.12–0.26	0.95–5.47

*bd, below detection; ^∗^indicates stations where SSS increased/decreased during the course of occupation on station; ^†^indicates stations where nutrient concentrations changed over the course of occupation on station.*

Dissolved inorganic phosphate and Si in the surface waters (<5 m) were inversely correlated with SSS in 2010 and 2011 (Supplementary Figures S1A,B). The relationship between DIN and DIP (N:P) in the surface waters of both cruises showed deviations from a 16:1 relationship (i.e., Redfield ratio), and the N:P in 2010 compared to 2011 were indicative of an increased N limitation (Supplementary Figures S1C,D). Moreover, the quasi-conservative tracer N^∗^, which can be used to estimate the distribution of N_2_ fixation (Gruber and Sarmiento, 1997), showed more positive values in areas of low DIN and DIP for both cruises (**Figure 2**).

Lower SSS were common in the western region (46° – 51° W, 5° – 8° N) of both cruises (**Figure 2**); one exception was station 23 of 2010 (54° W, 10° N). Warmer sea surface temperatures (SST) coincided with the lower SSS in 2010, whereas in 2011, higher SST (≈30°C) was measured to the north and northeast. In 2010, the low salinity lens varied 5–15 m and was enriched in DIP (0.21–0.59 μmols L^-1^) and Si (17.1–56.6 μmols L^-1^), however, DIN was often below detection (bd). In 2011, the low SSS lens penetrated the upper 12 m, and had a similar dissolved Si concentrations (19.3–42.1 μmols L^-1^) and larger range in DIP (0.18–1.02 μmols L^-1^) than in 2010. Similar to 2010, DIN was bd in the surface lens at the low SSS stations of 2011.

The mesohaline surface salinity stations of 2010 were localized in the northeast region (50° – 56° W, 5° – 11° N), while in 2011, mesohaline SSS was observed more broadly (**Figure 2**). Surface dissolved nutrient concentrations at the mesohaline SSS stations during both cruises were characterized as low in DIP (bd-0.60 μmols L^-1^), modest concentrations of Si (2.19–17.7 μmols L^-1^), and DIN was often bd.

Stations with surface salinities typical of oceanic conditions in 2010 and 2011 were located in the eastern region during both cruises and in addition to the north in 2011 (**Figure 2**). DIP and DIN were bd at most stations of 2010 with the exception of stations 6, 7, 20, and 27 where DIP measured 0.1–0.7 μmols L^-1^ and at station 27 the DIN measured 0.02 μmol L^-1^. Dissolved Si was measureable at all oceanic stations of 2010 and 2011 and ranged 0.78–5.99 μmol L^-1^. DIP and DIN were higher in concentration in the oceanic stations of 2011, where DIP measured as high as 0.26 μmol L^-1^ and DIN measured 0.11 μmol L^-1^ at station 26.

There were several low SSS stations that experienced fluctuations in their SSS and dissolved nutrient concentrations increased, decreased or remained unchanged while on station. An example was at stations 9 and 16 in 2010 and station 13 in 2011 where the SSS decreased by 3–6 PSU and DIP and Si each increased up to a twofold higher nutrient concentration. Only at station 9 did the DIN increase from bd to 0.27 μmol L^-1^.

### Qualitative Observations of Phytoplankton

The low SSS stations of 2010 (stations 4, 10, 14, 16, and 23) were composed of non-symbiotic diatoms; in particular, the surface sample at station 4 was largely composed of *Coscinodiscus* spp. (Supplementary Figure S2A), while observations from depth (21.6, 41.9, and 71.5 m) found higher densities of *Pseudo-nitzchia* spp. and *Skeletonema* spp. diatoms. The latter were also common at stations 10, 14, and 16 of 2010. Station 23, another low SSS, was dominated by non-symbiotic *Chaetoceros* spp. diatoms, and at 10 m, the high densities of *Chaetoceros* spp. were observed exuding a gelatinous matrix. Below the freshwater lens at several of the low SSS stations of 2010 (e.g., stations 4, 16, 23), symbiotic *H. hauckii* and *R. clevei* were observed.

Similar to the qPCR results (see below), high densities of the *H. hauckii-Richelia* symbioses were observed and limited to the meso and high SSS stations of 2010. Although highly abundant, the cell integrity of both symbiont and host varied greatly, such that both degraded cells (e.g., symbiont comprised of only a terminal heterocyst, host with reduced chloroplast biovolume or empty frustules) were observed mixed with fully ‘intact’ chain forming cells (e.g., 8–12 cells and < 50 cells) or recently divided cells (e.g., pairs of cells). Degraded or moribund looking *H. hauckii* symbioses were more common below the depth of maximum abundance and usually coincided with a higher abundance of *R. clevei-Richelia* symbioses with good cell integrity. The latter was most obvious at stations 5, 6, 7, and 20. Consistent with a poor detection by qPCR, the *R. clevei-Richelia* symbiosis was rarely observed during the 2011 cruise. The *H. membranaceus-Richelia* symbioses was less common on the 2010 cruise with the exception of station 1 where short chains were observed and the *Richelia* had no vegetative cells (Supplementary Figure S2B), while on the 2011 cruise it was the dominant DDA albeit at low densities.

Cells similar in size and morphology to *Crocosphaera watsonii* (hereafter referred to as *C. watsonii*-like cells) were infrequently observed at low densities in 2010 (station 4, 5, 6, 7, 17) and in 2011 (station 13). At several stations, and typically at depths below 30 m, *Trichodesmium* spp. colonies and/or free-filaments were observed in a state of degradation reminiscent of viral lysis or as short trichomes (e.g., hormogonia; for example, at stations 4, 6, 7, 16, 20, 25, 27 in 2010). A few observations (stations 19, 20 in 2010) were noted of large *Trichodesmium* colonies embedded with radiolaria, dinoflagellates, diatoms, or other plankton, including DDAs (Supplementary Figure S2C). *Trichodesmium* was less common in 2011 and observed as mostly as free filaments.

Picocyanobacteria, or cells with similar morphology and cell diameters (>0.2 mm) to *Synechococcus*, and pico-eukaryotes were also observed, despite their smaller cell diameters than the pore size of the collection filter. Both populations were observed as free cells, attached to marine snow particles, as components of fecal pellets, and colonial picocyanobacteria were also frequently observed at depth (Supplementary Figures S2D,E). Densities were higher for the pico-plankton in the deeper depths (e.g., DCM), and in particular at station 20 of 2010 it appeared to be ‘rainfall’ of *Synechococcus* (Supplementary Figure S2F).

### Abundance and Distribution of DDAs: Het-1 and Het-2

In 2010, the het-2 was highly abundant (detected in 76%, 73 of 96 samples) at all stations sampled with the exception of the low SSS stations (**Figure 2** and Supplementary Table S1). Modest densities (<10^2^ *nifH* copies L^-1^) for het-2 were detected at low SSS stations 4 and 16, but at 2 depths below the low SSS lens. Highest densities for het-2 were at the mesohaline followed by oceanic SSS stations (**Figure 2**). Although het-1 was present and co-varied in depth distribution to het-2, it was less abundant (bd-10^5^ *nifH* copies L^-1^) and only represented 0.06% of the total *nifH* copies detected compared to the 99.94% representative of het-2. Het-1 consistently co-occurred with het-2, and unlike het-2, the abundances were higher at the oceanic SSS compared to the mesohaline SSS stations. Interestingly, the average depth of maximum abundance for het-1 and het-2 were consistently parallel, and shallower at the oceanic SSS stations (8 m) compared to the mesohaline and low SSS stations (20.2 and 20 m, respectively).

In 2011, het-1 abundance was bd in all samples assayed. Het-2 was present, although at a lower detection (40%, 23 of 57 samples assayed) and limited to the mesohaline SSS stations; maximum abundances never exceeded 10^3^ *nifH* copies L^-1^. Given the limited number of samples taken (e.g., one low and one high SSS) and the low detection of het-2 in 2011, our comparison to 2010 is restricted. Unlike the 2010 expedition, the depth of maximum abundance varied greatly for het-2, from the surface (station 25) to 70 m (station 21) (Supplementary Table S1).

### Piecewise SEM

Given the primary interest of applying the SEM model was to predict the influence a parameter or sets of conditions have on DDA abundances, we summarize only variables that were significantly directly/indirectly affecting het-1 or het-2 abundance. Thus, we exclude the majority of influence of the environmental parameters on each other and any cascading effects in the system.

**FIGURE 3:**
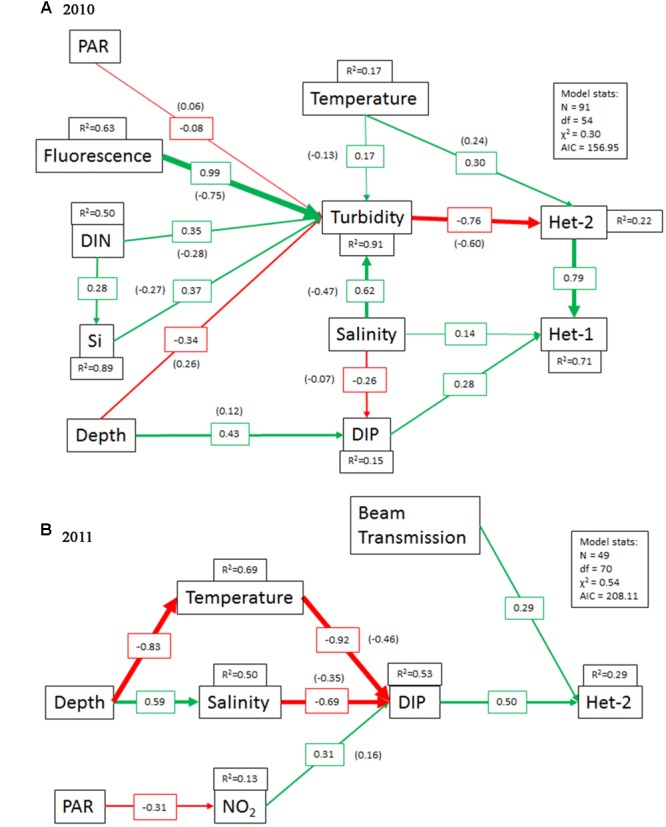
**Piecewise SEM models of environmental parameters as predictors of het-1 and het-2 *nifH* gene copy abundances in (A)** 2010 and **(B)** 2011. Arrows represent the unidirectional relationships among the parameters and only predictor variables, which either directly or indirectly effect the het groups, are shown. Positive and negative paths (*p* < 0.05) are shown as green and red arrows, respectively, with the thickness of the paths (arrows) scaled to the magnitude of the path strength. Path strengths are designated on arrows and variables lacking *R*^2^ values acted only as predictors. Values in parentheses are the indirect effects strength on either het-1 or het-2.

Overall, the SEMs were adequate fits to the data in both 2010 (χ^2^= 58.95, *p* = 0.30) and 2011 (χ^2^ = 68.11, *p* = 0.54) (**Figure 3**). Turbidity was the most significant parameter (-0.76, *p* < 0.0005), directly influencing the abundance of het-2 in 2010 (**Figure 3A**). Moreover turbidity mediated the indirect and negative effects of a number of other parameters (DIN, Si, fluorescence, salinity and temperature: -0.28, *p* < 0.001, -0.27, *p* < 0.0001, -0.75, *p* < 0.0001, -0.47, *p* < 0.0001, -0.13, *p* < 0.001, respectively) on het-2 abundance and in addition, had an indirect negative effect on het-1 (-0.60, *p* < 0.0005) mediated through het-2 abundance. Het-1 abundance was directly affected by both salinity (0.14, *p* < 0.05) and DIP concentration (0.28, *p* < 0.0001). One of the strongest relationships identified in the 2010 dataset was the positive direct effect het-2 abundance had on het-1 abundance (0.79, *p* < 0.0001).

The SEM model applied to the 2011 dataset identified a different set of conditions and decreased number of paths influencing the het-2 abundance (**Figure 3B**). DIP was the most prominent factor positively (0.50, *p* < 0.02) influencing het-2 abundance. Moreover, DIP mediated the indirect and negative effects of temperature (-0.46, *p* < 0.0001) and salinity (-0.35, *p* < 0.0001) on het-2 abundance. Nitrite concentration was also indirectly, but positively influencing het-2 abundance (0.16, *p* < 0.005) and was mediated through DIP. Beam transmission was identified as a positive variable on het-2 abundance in 2011 although the relationship was direct it was less robust (0.29, *p* < 0.03).

## Discussion

The WTNA is a highly dynamic region of the North Atlantic Ocean and is an ideal location to study the influence of riverine and atmospheric inputs on the biological community composition and their activity. Earlier studies in the WTNA have reported the influence of the AR and aeolian dust deposition on a variety of parameters related to the N cycle, e.g., DIN (Ryther et al., 1967; DeMaster and Pope, 1996; DeMaster and Aller, 2001), trace metal concentrations (Boyle et al., 1977; Chen and Siefert, 2004; Kaufman et al., 2005; Bergquist and Boyle, 2006), the presence and distribution of N_2_ fixing populations, including symbiotic diatoms (Villareal, 1994; Carpenter et al., 1999; Foster et al., 2007; Subramaniam et al., 2008; Goebel et al., 2010; Turk-Kubo et al., 2012; Goes et al., 2014; Hilton et al., 2015; Zelinski et al., 2016), carbon export (Cooley et al., 2007; Subramaniam et al., 2008; Yeung et al., 2012), and N_2_ and C fixation (Carpenter et al., 1999; Montoya et al., 2002; Sohm et al., 2011; Fernández-Castro et al., 2015). Given the new N supplied by symbiotic diazotrophy in the WTNA and in other regions of the World’s Ocean (e.g., N. Pacific; Venrick, 1974; Karl et al., 2012) fuels the surrounding community (biological pump) and is thought to support an efficient C export to the deep (Cooley and Yager, 2006; Cooley et al., 2007; Subramaniam et al., 2008; Yeung et al., 2012), determining the causal relationships between abundance and measured variables is of primary interest.

### Amazon River Impact on Hydrography and Phytoplankton Community Composition

Similar to earlier work in the WTNA, the influence and extent of the AR plume was distinguishable by decreased surface salinities during both cruises. Monthly MODIS (or Moderate Resolution Imaging Spectroradiometer) composites showed a broad influence on surface chl *a* and entrainment of the plume in the North Brazil current (NBC) and flow northwest toward the Caribbean Sea in 2010, while the composite from October 2011 showed evidence of the seasonal and narrow retroflection (Muller-Karger et al., 1988) at approximately 10°N where surface waters flowed easterly (Supplementary Figure S3). Consistent with earlier investigations is the immediate increase in chl *a*, and subsequent distinct community composition of a non-symbiotic diatom bloom in the AR plume waters. Whereas the surface abundances of both DDAs in 2010 appeared to increase with the plume’s entrainment northwest, in 2011, DDAs rarely exceeded background densities (e.g., <10^2^ *nifH* copies L^-1^). Moreover, only het-2 was detected by qPCR in 2011 and appeared less affected by the plume’s directional flow or the plume was overall less influential on the DDA abundances.

The dissolved nutrient concentrations measured during our expeditions were similar to previous investigations in the WTNA (Carpenter et al., 1999; Foster et al., 2007; Subramaniam et al., 2008; Goebel et al., 2010). Surface DIN was not correlated with SSS for either cruise, and was often below detection. Moreover, in 2010, DIN deficits were also coincident with low maximum quantum yield measurements (e.g., Fv/Fm) indicative of nutrient stress, including measurements made at the low SSS, or plume stations and in areas of high DDA densities (e.g., Fv/Fm = 0-0.2) (Goes et al., 2014). Given that DIN is not limiting for the DDAs due to the diazotrophic symbiont, other stressors or limitation must have influence over the DDA cell abundances and cell activity. Moreover, our microscopy observations of cell integrity of both symbiont and host were also indicative of non-optimal conditions at many of the high DDA abundance stations.

Both DIP and Si in the surface waters were inversely correlated with SSS during both expeditions suggesting the AR as major source of DIP and Si. Similar relationships between SSS and Si have been reported in the surface waters near Barbados in the Caribbean Sea (Steven and Brooks, 1972) and near Puerto Rico (Froelich et al., 1978). Despite measurably high concentrations of Si even in the low SSS lens, Goes et al. (2014) claimed Si deficiency throughout the 2010 cruise, especially upstream of the plume which was coincident with lower Fv/Fm measures and maximum abundance of non-symbiotic diatoms, many of which possess large cell diameters (*Coscinodiscus, Chaetoceros*) and thus higher Si requirements. A higher expression of silicon transporters was also identified in a metatranscriptomic study of the 2010 expedition (Zelinski et al., 2016) in the outer plume, which was coincident with the higher DDA bloom densities. Despite similar surface Si concentrations in 2011 and slightly elevated DIP concentrations, neither DDA reached high densities in 2011.

Others have reported evidence of a fresh water lens distant from the AR mouth with unique chemical and phytoplankton signatures (Borstad, 1982a,b) such as the early observations made in the Guinea Current (10°N) of the NBC (Ryther et al., 1967; Cochrane, 1969; Gibbs, 1970). These lenses, which are sometimes referred to as meanders, originate from the AR and carry distinct phytoplankton populations (Borstad, 1982a,b). The meanders can be 300 km in diameter and 20 to 50 m deep with salinity anomalies up to 9 PSU (Flagg et al., 1986). Notably, several stations in both 2010 and 2011 that were occupied for longer periods of time had considerable changes in salinity and dissolved nutrient concentrations within a short period of time. For example, stations 9 and 16 in 2010 experienced decreases in the SSS (4-6 PSU) with relatively sharp increases in dissolved nutrients (e.g., twofold increase in DIP and Si) within hours (e.g., 2.5 and 7 h, respectively) while abundances of *Richelia* remained relatively stable below the surface (8–16 m). However, in the immediate surface at these two stations, the abundance of *Richelia* associated with *H. hauckii* decreased strongly from 1.27 × 10^4^ *nifH* copies L^-1^ to near the detection limit (dnq). Both stations (9 and 16) are near one another (∼898 km) and on a northwest trajectory from the AR source. The decreasing salinity and increasing nutrients suggests a possible influence of a less saline AR meander, however, it appears that the near surface symbiotic diatom populations were either displaced by the meander and/or negatively influenced by its presence.

In 2010, warmer SST were localized to the stations of low SSS, which is also indicative of the AR influence. In contrast, the increased SST observed in 2011 was localized to oceanic stations far from the AR and therefore more suggestive of higher SST caused by increased stratification common to the open ocean. Moreover, the stations in 2011 with the highest SST also had the least dissolved nutrients (DIN, DIP, and Si) also indicative of decreased mixing from below and strong stratification. Thus, hydrographic conditions in the WTNA largely derived from the AR, favor DDAs, and in particular het-2 and *Hemiaulus* spp. hosts.

### Drivers of DDA Abundances and Distributions in the WTNA

Abundances of het-1 and het-2 from both cruises corroborate and complement a number of earlier studies in the North Atlantic (Villareal, 1994; Carpenter et al., 1999; Foster et al., 2007; Subramaniam et al., 2008; Goebel et al., 2010; Ratten et al., 2015). Although collections were from different years and few studies use similar units for reporting abundances, the combined earlier datasets represent a near complete yearly investigation of DDA abundance and distribution in the North Atlantic, and also underscore the widespread and persistent distribution of DDAs, in particular the *Hemialus-Richelia* symbiosis throughout the year in the WTNA (Supplementary Table S3).

In spite of different seasonal occurrences, the blooms of the *H. hauckii-Richelia* symbioses in the WTNA have a few common observed hydrographic features that seem to prime the region for high densities. For example, highest abundances are consistently recorded at mesohaline SSS, while the salinity and temperature at the depth of maximum abundance vary greatly from 31.4 to 36.17 PSU (mesohaline-oceanic) and 25.7–28.5°C, respectively. High densities of het-1 and het-2 have been reported in other less oceanic habitats with similar salinity and temperature optima, e.g., New Caledonia coast (Turk-Kubo et al., 2015); North American Coast (Ratten et al., 2015); Congo and Niger Plumes (Foster et al., 2009); S. China Sea (Bombar et al., 2011). Wider ranges in temperature optima appear to be more the norm (Moisander et al., 2010), however, unlike the het groups, other diazotrophs, e.g., *Trichodesmium* spp., have less of a range in salinity optima (e.g., 34–36 PSU).

Most blooms in the WTNA have been reported from early spring and are within the same range as the densities reported here in May–June of 2010. Moreover, maximum abundances in mesohaline SSS are often in the subsurface (20–25 m), while depth maxima for DDAs appear in the near surface at the oceanic stations, potentially favored by higher light intensities. Our correlation matrix (Spearman’s rho) also identified several highly significant (*p* < 0.001) relationships between het-2 (and het-1) abundance favoring higher light intensity and quality, including PAR and beam transmission (Supplementary Table S2). Similar correlations between PAR and het-1 and het-2 abundance was determined in a dataset from the SW Pacific in the Melanesian archipelago, where het-1 maximum abundance (15 m) was similar to that reported here in the subsurface (Stenegren et al., unpublished).

Waters depleted in DIN, but measurable DIP (0.04–0.13) and dissolved Si ranging from 1.0 to 10.7 μmol L^-1^ also appears to favor high abundances of DDAs in the WTNA (Supplementary Table S3). The higher range of DIP and Si is consistent with expected higher seasonal outflow of the AR, and is consistent with reports from early spring (Supplementary Table S3). In fact, the 2010 bloom reported here occurred during the maximum discharge of the AR for 2010 (Yeung et al., 2012). The low to background densities of the DDAs in 2011, when nutrient concentrations were seemingly similar to 2010, suggests that there is a seasonal pre-requisite for early dry season, however, similarly high abundances (>10^6^ cells m^2^) for the *H. hauckii-Richelia* symbioses have been reported in winter (Feb 2001) and fall (October–November 1996) (Carpenter et al., 1999; Subramaniam et al., 2008). Therefore, it seems that the high abundances for the DDAs, and *Hemiaulus-Richelia* in particular are not directly linked to a season or single environmental condition, but rather large ranges in salinities (mesohaline to oceanic), temperatures, irradiances, and nutrient concentrations. The latter highlights the difficulty in identifying a single parameter that drives DDA abundance.

Draft genomes for two of the *Richelia* strains (het-2) which associate with *Hemiaulus* spp. and the *Richelia* symbiont of *R. clevei* (het-1) have been sequenced and their genomic capacity indicates that *Richelia* cannot utilize a number of DIN sources (e.g., nitrate, nitrite, urea) and lacks ammonium and urea transporters (Hilton et al., 2013). The het-2 strains, in addition, lack alkaline phosphatase, *phoA* (Foster, unpublished). Alkaline phosphatase is used to cleave free phosphate groups through the hydrolysis of phosphomonoesters (Kuenzler and Perras, 1965) and is indicative of P stress. A closer comparison of the genes for P acquisition in the het strains identifies a number of disparities. For example, the het-1 symbiont draft genome contains in addition to *phoA*, several genes for dissolved organic P (DOP) acquisition and transport (e.g., *phnD*, phosphonate binding; *phoD*, phosphodiesterase; *ppK, ppX* polyphosphate kinase). Thus, the genome content of the *Richelia* het-2 symbiont requires measurable DIP, while the *Richelia* het-1 has a broader genetic repertoire for scavenging P and, perhaps can alleviate P limitation by utilizing DOP. Although we have no information on DOP concentrations, higher abundances of het-1 were enumerated and perhaps favored at the oceanic stations, areas with the lowest DIP and DIN concentrations. Evidence of DOP positively influencing abundance, and *nifH* gene transcription by het-1 was recently reported in a mesocosm experiment in the subtropical eastern North Atlantic (Meyer et al., 2016). It should be noted that the *Richelia* genomes are draft, and therefore lacking a gene or pathway could be a result of incomplete genome sequencing. The differences in the het strains genome content does highlight how the DDA symbionts differ in potential resources which could easily drive their distribution, abundances, and niche adaptation. Similar adaptations have been identified in other diazotrophs (Dyrhman and Haley, 2006).

### Application of Piecewise SEM to Predict the Drivers of DDA Abundances in the WTNA

Piecewise SEM (hereafter referred to as SEM) is still new to the field (Lefcheck, 2016), however, it has been successfully applied in a diverse suite of studies to determine the links between ecosystem multifunctionality and biodiversity, the role of biodiversity on top-down controls, and the role of multitrophic functional diversity on estuarine ecosystem function (Duffy et al., 2015; Jing et al., 2015; Lefcheck and Duffy, 2015). SEMs are often referred to as probabilistic models where a number of predictor and response variables are connected within a network of direct, indirect and cascading effects (Lefcheck, 2016). Moreover, the SEM differ from more traditional model approaches by incorporating hypothesized causality between variables, and allowing variables to be both predictor and response variables in the same model (Lefcheck, 2016). The causality is based on previous knowledge of the investigated system, meaning that the SEM is built on informed choices and hypotheses.

Historically, blooms of the *Hemiaulus-Richelia* symbioses are common in the WTNA, and correlated with seasonal discharge and SSS (Subramaniam et al., 2008). Thus, we expected to find correlations between het-2 abundance and variables indicative of the AR. Second, we hypothesized that high abundances of one DDA, e.g., het-2 as observed in 2010, would have a negative impact on the other DDA since resources could have become limiting or competitive. Finally, we hypothesized that there are conditions or sets of conditions that favor one DDA over the other given that host diatoms differ (genus, size), as do the symbiont strains (e.g., trichome length, location, diversity and genome content).

Contrary to our predictions and previous findings that DDA abundance, in particular *H. hauckii-Richelia* symbioses, is largely influenced by salinity and localized to regions of fast DIN utilization relative to DIP, the SEM did not find significant direct interaction between salinity or the latter nutrients and het-2 abundance. Despite high abundances quantified by qPCR, our observations of cell integrity for the *H. hauckii-Richelia* symbioses suggested that as a whole, the populations appeared senescent. The latter may explain the poor correlation between salinity and/or nutrients and het-2 abundance. In fact, most often mixed populations of moribund and healthy cells were observed. However, if we consider the indirect effects of nutrients and salinity, the SEM model did in fact identify that both parameters were indirectly influencing the het-2 abundance mediated through turbidity. Moreover, salinity and DIP directly influenced het-1 abundance. Thus, the SEM model supported our initial hypothesis that the AR is a predictor of DDA abundance.

Turbidity is also an important parameter influencing species abundance and activity and related to the AR plume. Likewise, turbidity was identified in the 2010 SEM as a robust negative parameter directly and indirectly influencing het-2 and het-1 abundances, respectively. The AR discharge is equally high in particulates and colored dissolved organic matter (CDOM), as in dissolved nutrients, and as such limits light availability for photosynthesis (Nittrouer et al., 1986; Smith and DeMaster, 1996; Del Vecchio and Subramaniam, 2004). In the low SSS and mesohaline SSS with higher particulate loads, DDAs reside below the plume, or penetrate deeper depths favored by sufficient light for photosynthesis. Indirectly, DIN and Si negatively impacted het-2 through turbidity (predictor), which is consistent with the findings of Goes et al. (2014) that the phytoplankton community structure during the 2010 cruise was in a Si deficiency and largely controlled by salinity and nutrient limitation. Hence, much of the environmental influence on the DDAs seems to be due to the indirect effects of turbidity, or the AR plume, mediating light quality and nutrient concentrations.

One of the more surprising outcomes of the SEM model was the robust positive influence (0.79, *p* < 0.0001; *R*^2^= 0.71) on het-2 had on het-1 abundance. Het-1 and het-2 co-varied with depth; similar distributions and strong correlations between the two symbiotic strain abundances have been previously documented in diverse habitats, including the Western Tropical South Pacific (WTSP), South China Sea, North Pacific Gyre, and earlier in the WTNA (Stenegren et al., unpublished). However, in correlation analyses, the direction of influence cannot be resolved, as in our SEM model. The specific mechanism of het-2 influence on het-1 abundance remains unknown.

Still unresolved is why the *Hemiaulus-Richelia* symbioses tend to dominate in this region of the ocean rather than the *Rhizosolenia-Richelia* symbioses. The host taxonomy and morphological characters differ greatly, e.g., cell diameter (Sournia, 1986; Villareal, 1992), which equates to different Si requirements. The difference in host frustule dimension is one obvious precondition that could drive differences in parameter influence. Si was not directly influencing het-1 or het-2 abundance, however, Si was considered an indirect negative predictor of het-2 abundance in 2010. The latter supports the conclusion by Goes et al. (2014) of an efficient Si drawdown mediated by both non-symbiotic and DDA populations leading to an overall Si deficiency. Moreover, the SEM model shows direct positive effect of salinity on het-1 and an indirect negative effect of salinity on het-2. Therefore, het-1 seems to thrive more at higher salinities as detected by our qPCR, and explains the higher abundances of het-1 over het-2 in the open ocean habitats (Church et al., 2008; Stenegren et al., unpublished).

In conclusion, our study represents the first application of SEM to determine the direction of environmental parameter impact on DDA abundances, which is a departure from the commonly used univariate statistics (e.g., correlation based analyses). The resemblance in our SEM model predictions of the two cruises (bloom vs. background densities) demonstrated that several environmental parameters (temperature, salinity, turbidity, and dissolved nutrients) acted directly and in concert to influence DDA abundances in a highly dynamic ecosystem. The SEM model output also establishes a framework of new and testable hypotheses by lab based experiments and/or similar application of the SEM to other datasets.

## Conclusion

Our study showed and corroborated with earlier works that the AR plume has a strong impact on the hydrography, and community composition in the WTNA. In particular, the *Hemiaulus-Richelia* symbiosis is favored in the WTNA when warm (>28°C) mesohaline waters are devoid of DIN, but require measurable concentrations of DIP and silicate to proliferate. Lastly, our SEM model confirmed our observations of complex interactions between environmental parameters in the ocean and how they impact the het-1 and het-2, directly and indirectly. Unexpected, and still unexplained, was evidence for a positive direct influence of het-2 abundance on het-1. A next obvious and applicable step of the SEM model is its application to other diazotrophs and regions of the world’s oceans, in order to predict how diazotrophic populations are distributed and potentially function.

## Author Contributions

RF designed the study, made the sample collections, and performed the microscopy observations. CB and MS designed, optimized and implemented the SEM model. S-SD and CP extracted the DNA and ran the qPCR reactions. JM collected, measured, and provided nutrient data and PY provided ship-time and all hydrographic parameters from expeditions. The paper was written by RF, MS, and CB and reviewed by all co-authors.

## Conflict of Interest Statement

The authors declare that the research was conducted in the absence of any commercial or financial relationships that could be construed as a potential conflict of interest.

## Footnotes

1 https://www.R-project.org/
