# Supplementary material for: Piecewise Structural Equation Model (SEM) Disentangles the Environmental Conditions Favoring Diatom Diazotroph Associations (DDAs) in the Western Tropical North Atlantic (WTNA)
Source: Front Microbiol. 2017 May 9;8:810. doi: 10.3389/fmicb.2017.00810 (PMC5423296; doi:10.3389/fmicb.2017.00810)
Supplement: Supplementary file 2 [file Data_Sheet_1.docx]

Suppl. Figure 1


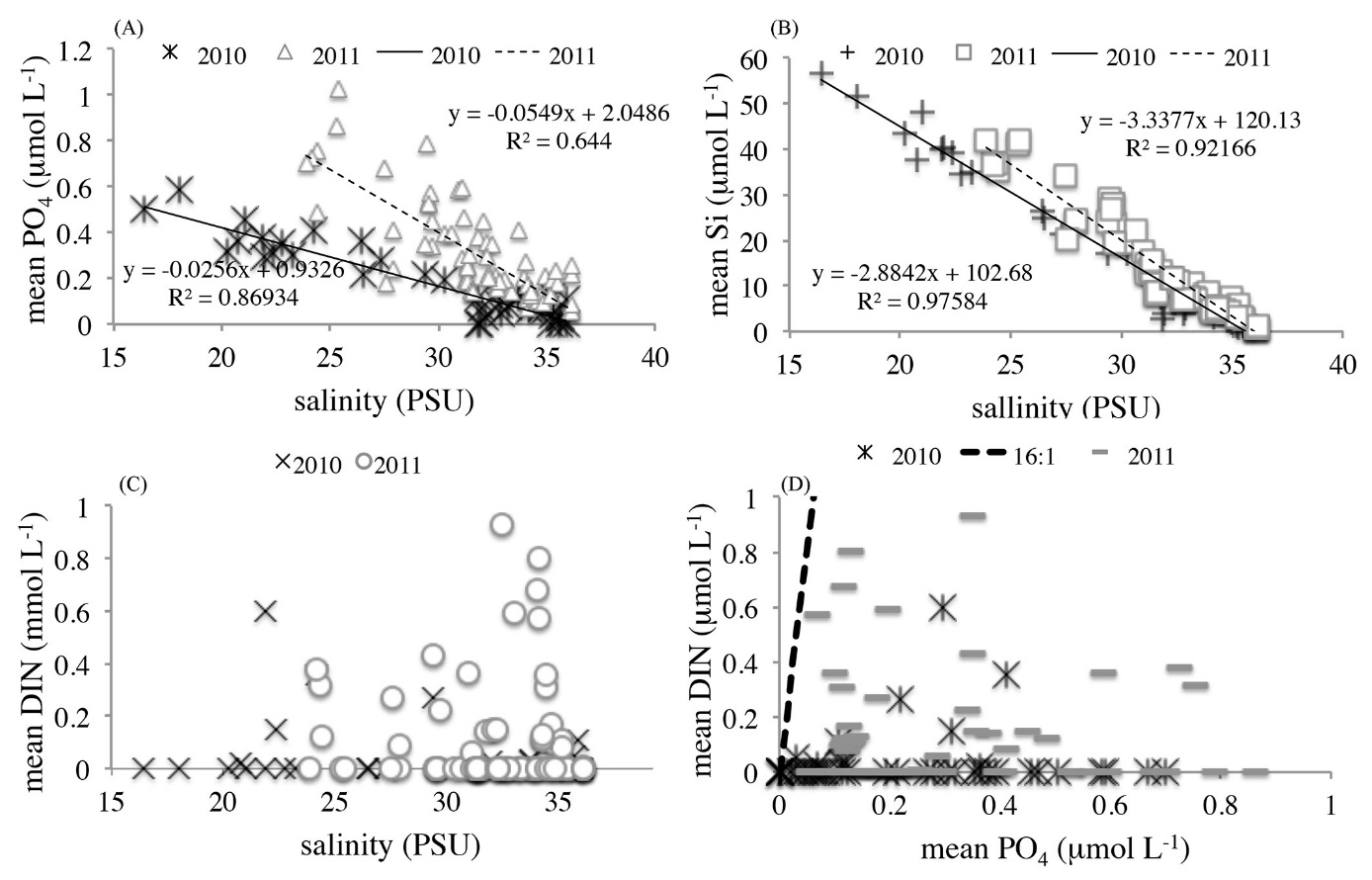


Suppl. Figure 1. (A) Sea surface (< 5m) dissolved phosphate (PO_4_) (B) silicate (Si), and (C) inorganic nitrogen (nitrate and nitrite) concentrations as a function of sea surface salinity for the 2010 and 2011 expedition to the WTNA. All stations for both expeditions are plotted with the exception of station 27 in plot A. Linear regressions are shown for A and B. (D) DIN versus PO_4_ concentration in sea surface and the dashed line indicates an N:P of 16.

Suppl. Figure 2.


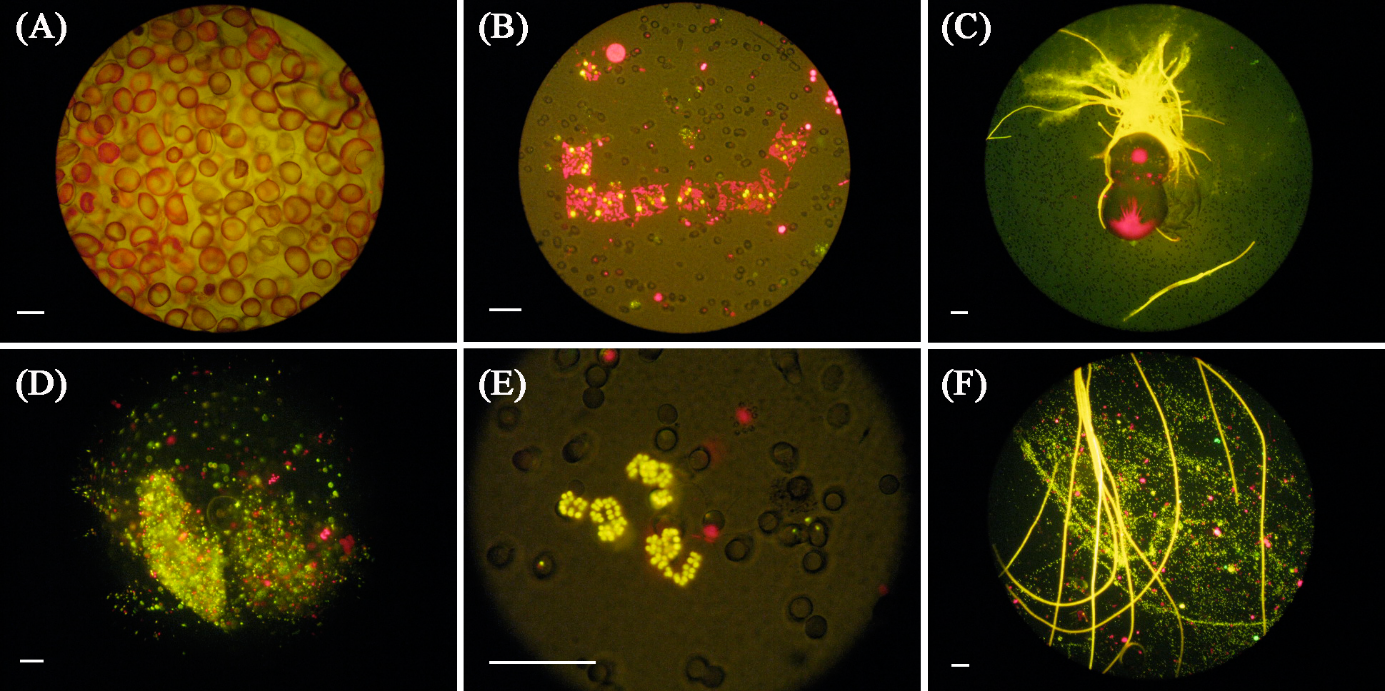


Suppl. Figure 2. Epifluorescent images of the phytoplankton community observed during the 2010 expeditions to the WTNA. Blue excitation micrographs taken in the field of (A) *Coscinodiscus* spp. diatoms from station 4; (B) a chain of symbiotic *H. membranaceus* diatoms shows a dense chloroplast (red) in the diatom host and cassociated *R. intracellularis* trichomes (yellow spheres) composed of only terminal heterocysts; (C) a *Trichodesmium* spp. colony in a state of degradation and embedded with two *Pyrocystis* spp. dinoflagellates; (D) a aggregate or fecal pellet composed of picocyanobacteria and picoeukaryotes; (E) several small colonies picocyanobacterial cells held together in an extracellular matrix; (F) the ‘rainfall’ of picocyanobacteria and free trichomes of *Trichodesmium* spp. observed at station 20. Scale bars are approximations of 50 μm.

Suppl. Figure 3


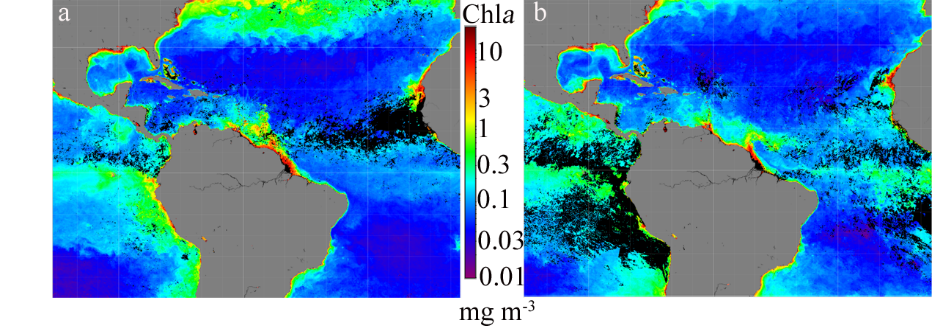


Suppl. Figure 3. Monthly MODIS composite for the two cruise expeditions to the WTNA near the Amazon River Plume. A. May-June 2010 B. Sept-Oct. 2011
